# Assessing the Significance of Conserved Genomic Aberrations Using High Resolution Genomic Microarrays

**DOI:** 10.1371/journal.pgen.0030143

**Published:** 2007-08-24

**Authors:** Mitchell Guttman, Carolyn Mies, Katarzyna Dudycz-Sulicz, Sharon J Diskin, Don A Baldwin, Christian J Stoeckert, Gregory R Grant

**Affiliations:** 1 Penn Center for Bioinformatics, University of Pennsylvania, Philadelphia, Pennsylvania, United States of America; 2 Department of Pathology and Laboratory Medicine, University of Pennsylvania School of Medicine, Philadelphia, Pennsylvania, United States of America; 3 Division of Oncology, Children's Hospital of Philadelphia, Philadelphia, Pennsylvania, United States of America; 4 Penn Microarray Facility, University of Pennsylvania, Philadelphia, Pennsylvania, United States of America; 5 Department of Genetics. University of Pennsylvania School of Medicine, Philadelphia, Pennsylvania, United States of America; Jackson Laboratory, United States of America

## Abstract

Genomic aberrations recurrent in a particular cancer type can be important prognostic markers for tumor progression. Typically in early tumorigenesis, cells incur a breakdown of the DNA replication machinery that results in an accumulation of genomic aberrations in the form of duplications, deletions, translocations, and other genomic alterations. Microarray methods allow for finer mapping of these aberrations than has previously been possible; however, data processing and analysis methods have not taken full advantage of this higher resolution. Attention has primarily been given to analysis on the single sample level, where multiple adjacent probes are necessarily used as replicates for the local region containing their target sequences. However, regions of concordant aberration can be short enough to be detected by only one, or very few, array elements. We describe a method called Multiple Sample Analysis for assessing the significance of concordant genomic aberrations across multiple experiments that does not require a-priori definition of aberration calls for each sample. If there are multiple samples, representing a class, then by exploiting the replication across samples our method can detect concordant aberrations at much higher resolution than can be derived from current single sample approaches. Additionally, this method provides a meaningful approach to addressing population-based questions such as determining important regions for a cancer subtype of interest or determining regions of copy number variation in a population. Multiple Sample Analysis also provides single sample aberration calls in the locations of significant concordance, producing high resolution calls per sample, in concordant regions**.** The approach is demonstrated on a dataset representing a challenging but important resource: breast tumors that have been formalin-fixed, paraffin-embedded, archived, and subsequently UV-laser capture microdissected and hybridized to two-channel BAC arrays using an amplification protocol. We demonstrate the accurate detection on simulated data, and on real datasets involving known regions of aberration within subtypes of breast cancer at a resolution consistent with that of the array. Similarly, we apply our method to previously published datasets, including a 250K SNP array, and verify known results as well as detect novel regions of concordant aberration. The algorithm has been fully implemented and tested and is freely available as a Java application at http://www.cbil.upenn.edu/MSA.

## Author Summary

Cancer is a genetic disease caused by genomic mutations that confer an increased ability to proliferate and survive in a specific environment. It is now known that many regions of genomic DNA are deleted or amplified in specific cancer types. These aberrations are believed to occur randomly in the genome. If these aberrations overlap more than would be expected by chance across individual occurrences of the cancer this suggests a selective pressure on this aberration. These conserved aberrations likely represent regions that are important for the development, progression, and survival of a specific cancer type in its environment. We present a method for identifying these conserved aberrations within a class of samples. The applications for this method include accurate high resolution mapping of aberrations characteristic of cancer subtypes as well as other genetic diseases and determination of conserved copy number variations in the population. With the use of high resolution microarray methods we have profiled different tumor types. We have been able to create high resolution profiles of conserved aberrations in specific cancer types. These conserved aberrations are prime targets for cancer therapies and many of these regions have already been used to develop effective cancer therapeutics.

## Introduction

In cancer cells, aberrations can turn on or off various pathways necessary for tumor development and survival [1]. Array comparative genomic hybridization (aCGH) is a highly parallel microarray-based method for detecting DNA copy number aberrations. aCGH detects genomic aberrations at a higher resolution than previous methods including metaphase chromosome–based CGH ([2,3], reviewed in [4,5]), and has proven to be a powerful tool for determining genomic aberrations of interest in various cancer types [6–8]. Similarly, this technology is quickly becoming widely used to characterize the genomic aberrations in various genetic disorders ([9,10] reviewed in [11]).

The analysis of new high resolution CGH data has proven challenging because most of the technical issues present in microarray gene expression analysis are also present in aCGH, as well as some new CGH-specific challenges. The most fundamental problem is to transform raw microarray data into the most accurate copy-number calls at the highest resolution possible (see [12] for review). This is known as the single sample problem, and there have been numerous publications suggesting approaches to this problem, including hidden Markov models [13], Circular Binary Segmentation (CBS) [14], and wavelets [15]. The common theme of these methods is that they attempt to find aberrant segments in the genome by using neighboring probes as replicates to give evidence of aberration at proximal locations.

Such single sample approaches can significantly decrease the native resolution of the array and result in a loss of information because important aberrations can be short enough to be detected by only one, or very few, array elements. If only one array is being analyzed, or if one is interested in the aberrations that are unique to a given individual, then there is little choice but to use one of the single sample methods. However, when the goal is to find concordant aberrations across a class of samples, we can take a different approach. In the multiple sample case we can perform statistical tests for concordant signal across samples, for each array element individually. This allows multiple (class-specific) samples to provide replication for each array element individually, in order to control the error rates statistically. In this way, resolution can be as fine as the probe spacing allows. This approach allows for leveraging multiple samples to simultaneously increase the resolution and the power of the analysis. To date, few methods have attempted to address this multiple sample problem statistically [16–19].

### Methodology

Considering one experiment at a time, it is difficult to determine effective parameters to make single sample calls, because it is difficult to distinguish signal from noise when aberrations are small. Looking across multiple samples for consistent effects it becomes clearer what is concordant signal and what is noise. We will define concordant signal as any aberration that occurs at a given location in more samples than would be expected by chance, under a null model, using some reasonable statistic.

In order to assess the significance of concordant aberration from a set of samples given single sample aberration calls, we use a nonparametric approach based on the Significance Testing for Aberrant Copy number (STAC) algorithm [16,20], which provides permutation based concordance *p*-values for each location. A nonparametric approach is taken because the true distributions involved in aCGH data are not known, nor can they be reasonably estimated. Therefore, in order to avoid making unrealistic assumptions about the data that would be required in a generative model, we rely on standard permutation approaches to obtain *p*-values to assess significance [21]. The null hypothesis is: given the rate of aberration for each sample, the locations of the aberrations are independent from sample to sample. To date, all multiple sample statistical methods, including STAC, take as input a set of aberrant intervals for each sample. However, we generally do not know the single sample aberrations, or the optimal criterion at which to determine aberration regions for each sample. This introduces an element of arbitrariness into a STAC analysis in that there are many ways to make the single sample calls to prepare the input to STAC. Furthermore, it is not clear that there is an optimal criterion at which to make single sample calls from microarray intensities, as different structural aspects of the data and different levels of noise are observable at different sensitivities (i.e., thresholds that we use to make the calls), and any given one may miss important information. This is demonstrated below on real data. Multiple Sample Analysis (MSA) aims to capture as much information as possible by measuring significance across a range of parameter values, and merging the information, with attention to multiple-testing issues. This allows us to gain power in the concordance analysis while controlling the family-wise error rate (FWER) for multiple locations. A final aberration call is made for each sample, at each location of significant concordance, by using the parameters that resulted in a significant *p*-value at that location. The parameter cutoff for making an aberration call in the samples at a given location is therefore a function of location because signal-to-noise ratio (SNR) varies at each location, depending on the level of aberration, the hybridization affinity of the probe, spatial effects of the array, normalization, and other factors.

MSA provides high resolution mapping of aberrant regions and provides a statistically meaningful method of integration between experiments. MSA is not just a substitute at low resolution for the single sample approaches; it is a different way of approaching the problem, a way that can give more powerful information about the experiments and the samples of interest.

## Results

### Aberration Calls

There are several natural criteria by which to quantify the raw signal from an individual array element into an aberration call at that location. The simplest is a straightforward threshold cutoff for the sample/normal signal ratio. If the data were perfect then the cutoff of 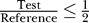 for loss and 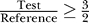 for gain would be sufficient. In practice, any criterion will offer a trade-off between true and false signal. If the null distribution of these ratios varies significantly from array element to array element, then a single cutoff can be conservative for some elements and liberal for others.

Many effects can introduce bias that will be difficult to distinguish from biological signal unless it is controlled for in the experimental design. Bias of two types can occur, across sample bias and within sample bias. Across sample bias can occur due to probe-specific hybridization, sequence bias, amplification bias, or many other probe-specific factors. In this case there will be a nonrandom distribution of observed “aberrations” when in fact there is no biological aberration. Within-sample bias can occur when contiguous regions on the chromosome are dependent for reasons other than biology, such as amplification bias causing contiguous regions to be over or under amplified. These aberrations have been noted before and were termed “local trends” by Olshen et al. [14]. We have similarly observed this effect when we employ an amplification protocol prior to hybridization. While our method would not be affected by within-sample bias, because it will be seen as noise in the null model, it will be affected by concordant bias, as would any multiple sample method. To address this issue, it might be necessary to perform a number of normal/normal hybridizations to estimate the normal/normal distributions individually for each array element. We define a normal/normal distribution as a distribution of normal cells hybridized and processed similarly to the real data. The criterion for each array element can then be based on the distribution for that element alone—for example, the standard deviation for each probe can be used as a cutoff for its corresponding element. We note that this effect can not be controlled for computationally due to the contiguous and concordant nature of many of these aberrations.

In all cases, we assume we have a criterion that is based on some kind of cutoff, and we are interested in assessing concordant signal across multiple samples based on varying this cutoff appropriately. Even when using a single sample approach such as CBS, one needs to define a cutoff parameter to determine amplification and deletion of regions of aberration. This step will be described more precisely below. For any fixed cutoff we test for significance by using the STAC algorithm [16], which provides permutation-based concordance *p*-values for each location, which are multiple testing corrected to control the FWER for the multiple locations being tested [21].

### STAC Algorithm

Given aberration regions in multiple samples, STAC defines two statistics to measure concordance, the “frequency” and the “footprint” [16]. For each statistic and each location a multiple testing corrected permutation *p*-value is computed, as described below. The frequency statistic measures the percent of samples with a given aberration at a given position. The footprint statistic measures tightness of alignment of a set of aberrant intervals that cover a given location, and is more sensitive than the frequency in most cases.

There are a few important aspects of the STAC algorithm that we take advantage of in our method. First, STAC provides *p*-values for concordance of aberration at each position. Second, the STAC *p*-values are multiple testing corrected across genomic positions to control the FWER. Third, the STAC footprint *p*-value takes into account the size of the region of aberration as well as the overall rate of aberration in the genome.

### Permuting the Data

**Figure 1:**
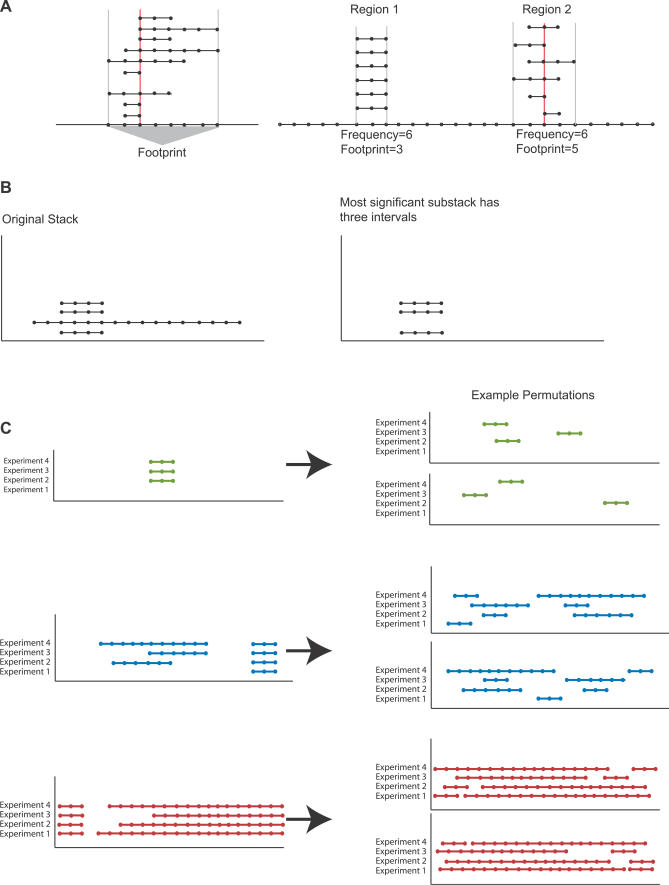
Illustration of Key Terms Used in the Description of the Analytical Method (A) The footprint is defined for a given stack as the vertical projection on the genome of all overlapping intervals. The footprint measures the tightness of the overlapping intervals within a given stack. The frequency measures the number of overlapping intervals over a given location. These two metrics are sensitive to different effects, as a region can have the same frequency but different footprints, such as Region 1 and Region 2, which share a frequency but have different footprints. (B) A stack contains substacks of sizes 2, …, *k* (the number of intervals in a given stack). An example of a stack and its most significant substack of size three is shown. (C) Sample permutations are illustrated on data where little is aberrant to data where most of the genome is aberrant. A given interval is permuted by randomly placing an interval in the genome rather than breaking up the positions within an interval. Each sample is permuted independently.

The permutation scheme moves each interval of aberration in each sample to a random location. Entire intervals are moved without breaking or resizing them in order to maintain the dependency between neighboring aberrant sites, while perturbing any alignment between samples. The goal of the permutation scheme is to maintain as much of the structure as possible in each sample while disrupting alignment between samples. An example of data and its sample permutations is shown schematically in Figure 1C.

### Frequency Statistic

**Figure 2:**
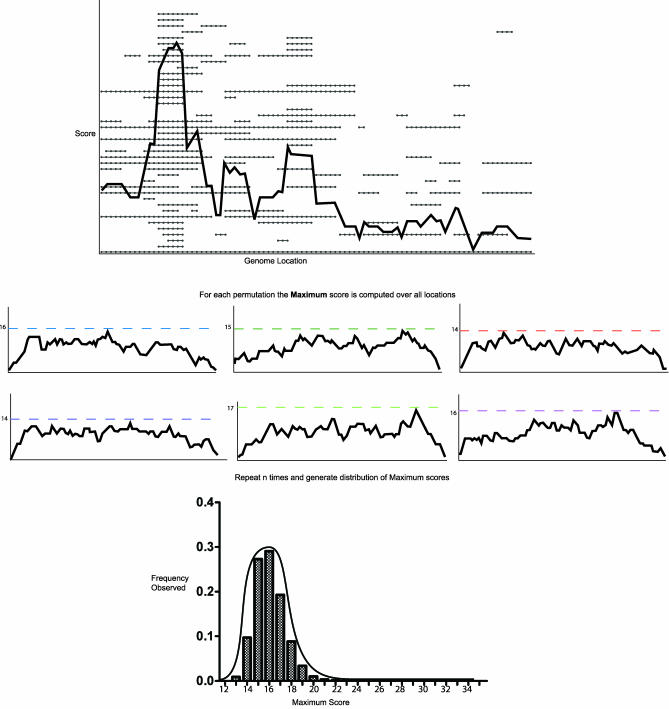
Calculating *p*-Values from Raw Data This figure illustrates how *p*-values are computed for the frequency statistic and the footprint statistic. The example tracks the frequency score but the argument is analogous for the footprint. We first begin with the raw data and calculate a score for each position. We then permute the data computing a maximum score for each permutation. We repeat the permutations *n* times, generating a distribution of the maximum observed score for each permutation. We then compare the score for each position on the genome to the distribution of the maximum score to compute multiple testing corrected *p*-values for each location.

The frequency (*Y_u_*) is the number of intervals that overlap a particular location *u,* where *u = 1 . . .L,* length of genome. Rather than drawing a threshold cutoff for making calls, which does not take into account the background rate of aberration or control the false-positive rate, a permutation test is performed. Given a permutation of the data, we calculate 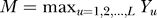 . A *p*-value is then obtained by comparing each observed *Y_u_* to the distribution of *M* (Figure 2). Since the distribution is of the maximum frequency over all locations, the *p*-value is multiple testing corrected.

**Figure 3:**
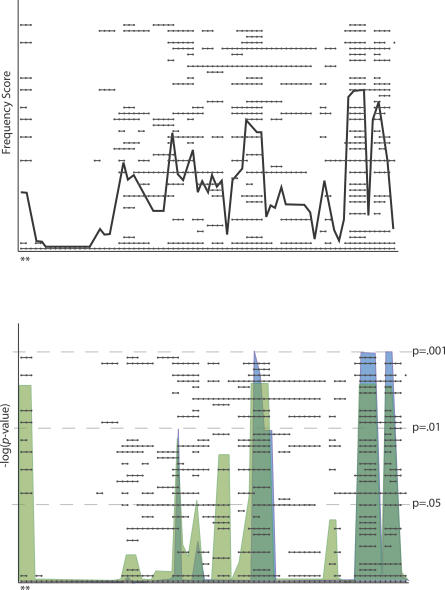
Illustrates How the Footprint Can Identify Regions That the Frequency Misses The starred region has a frequency that occurs under the permutation model frequently (frequency *p* = 1), yet the structure suggests a true aberration is present. The footprint statistic identifies this aberration as significant. This also illustrates the dependency between the footprint and frequency. Regions identified by the frequency are also identified by the footprint plus additional regions. The blue area tracks –log*(p)* for the frequency. The green area tracks –log(*p*) for the footprint. The gray dotted lines indicate different significance levels.

The frequency can fail to detect important regions of concordance within datasets because it fails to exploit the structure of the data and the intervals overlapping a location. For example, in Figure 1A, Region 1 and Region 2 have the same frequency but the frequency statistic will fail to detect any difference between them. In reality, the concordance of arrangement A suggests that true aberrations are more likely occurring at that location compared to arrangement B. Figure 3 illustrates in real data a location where the frequency is not significant in the permutation model but the alignment of the intervals suggests a real aberration. The footprint statistic is more sensitive to these effects.

### Footprint Statistic

A stack is defined as a set of intervals that lie over the same location. The location is called an “anchor point” of the stack. A stack contains at most one interval from each sample; however, it need not contain all intervals over a given location. If a stack has *n* intervals we refer to it as an *n*-stack. The footprint is defined for each stack and measures the length of the projection of a given stack onto the genome (Figure 1A). Any given stack contains many substacks. For example, a stack of four intervals contains four 3-stacks and six 2-stacks (Figure 1B).

To make the footprint comparable among stacks involving intervals of differing widths, it is normalized by the expected footprint: 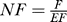 , where *EF* is the expected value of the footprint under the permutation model. This eliminates the bias that shorter intervals tend to have smaller footprints. In other words, stacks that are more tightly aligned tend to have smaller normalized footprints regardless of the lengths of the intervals involved in the stacks.

Additionally, long intervals can obscure the alignment of a stack over a location (Figure 1B). Therefore, in order to assess the significance of the footprint at a given location we look for tightly aligned substacks of the stack of all intervals anchored at the location. To assess significance, we perform a subset search to identify the minimum normalized footprint of all substacks over a given genomic location.

For a fixed stack *S* a *p*-value is assessed as follows. For each permutation of the data the smallest normalized footprint is determined over all stacks that have the same number of intervals as the stack in question. This provides a permutation *p*-value for the stack. A footprint-based “score” for a given location is then taken to be the minimum *p*-value of all stacks anchored at the location. The scores themselves cannot be taken as *p*-values because they are the minimum of many *p*-values. The significance level of each score is instead assessed via a second round of permutations, analogously to how the frequency *p*-values are assessed.

The quantities involved cannot typically be computed exactly because of the large number of possible substacks in the genome (figure 1B). Therefore, the minimum normalized footprint over each location is heuristically approximated.

### Subset Search and Run Time

In our implementation we use the algorithm as described in Grant et al. [20] and Diskin et al. [16]. However, we employ a modified search strategy that allows for a much faster approximation of the minimum normalized footprint over all possible subsets in the aberrant profiles.

STAC, as described by Diskin et al., runs at O(*M* ^4^ *L* ^3^) per permutation, where *M* is the number of samples and *L* is the number of locations in the genomic region being analyzed. This runtime is further affected by the constant *B,* which represents the search parameter introduced by Grant et al. and subsequently used by Diskin et al., *B* can only be regarded as a constant if the value of *B* is constant for all analyses. In reality, *B* must be significantly larger than *L* to ensure all positions are represented at least once in the smallest *B* stacks. Furthermore, the choice of *B* can change the results of the analysis significantly. As discussed in Diskin et al., as the parameter is raised, the global minimum is approached; however, the computational complexity increases rapidly with the size of *B.* Therefore, we would want to make sure that *B* is chosen as to make computation as accurate and efficient as possible.

**Figure 4:**
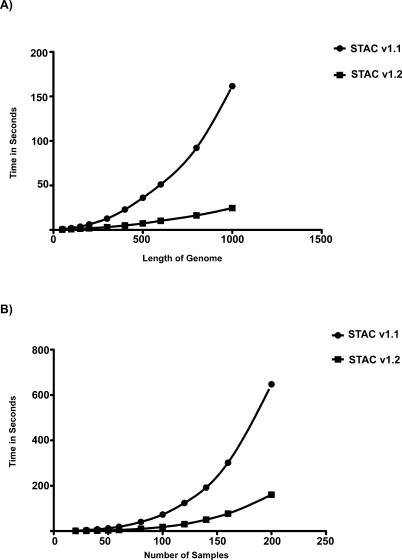
Run Time of the STAC Algorithm The time needed to run the STAC algorithm based on the original implementation and our new implementation. (A) Plots the run time as the length of the genome increases. (B) Plots the run time as the number of samples is increased. The numbers do not represent a typical dataset but rather a situation where every profile contains many aberrant intervals. For most real datasets the run time is significantly faster.

Our implementation differs from the original STAC algorithm in that the search phase is performed at each location separately which effectively reduces the search parameter to one. This reduces the computational complexity from O(*M* ^4^ *L* ^4^) to O(*M* ^2^ *L* ^2^) and eliminates the search parameter by changing the heuristic search algorithm for determining the minimum normalized footprint. At each anchor point *a* we estimate the smallest normalized footprint for stacks of size 2, 3, …, *M* by taking the smallest normalized footprint for step k and extending it into all possible *k* + 1 stacks anchored at the same location, and taking the one with the smallest normalized footprint. For each possible anchor point we have an array of minimum normalized footprints for 2, 3, …, *N*. We do this for all anchor points, which is at most *L,* the size of the genome, and take the global minimum to obtain the distributions used as in Diskin et al. [16]. Extensive testing against the original algorithm showed very little difference in reported *p*-values; however, the new method is significantly faster. A plot of actual computing time as a function of the length of the genome and the number of samples is shown in Figure 4. The optimized version, STAC 1.2, is available for download and the new search method is described in detail in the technical specifications.

### Data Processing

There are several considerations to make in practice. Some arrays have tighter distributions across all elements and as such require more liberal cutoffs to achieve the same amount of signal compared with other arrays that have broader distributions. To take this effect into account we have implemented scale normalization [22] to normalize between arrays. To the extent that this normalization causes us to be too liberal on some samples, it will not result in concordant false positives across multiple samples, so long as the noise is distributed in each sample independently. This is expected if concordant bias is properly controlled for, as discussed above. Regardless of what statistical methods are used to test for concordance, any concordant bias must be controlled for at the level of the experiment design.

### Selection of Cutoff Values

The sensitivity and specificity of any given cutoff depends on the rate of aberration of the unit of analysis, e.g., the entire genome, a single chromosome, or a chromosome arm. In most cases, we expect the rate of aberration to be different between different chromosome arms, because this has been observed across a wide variety of tumor types. In this case, the sensitivity of the analysis will be higher when performed separately on these units. In the examples provided, the typical unit of analysis is the chromosome arm. In other specific cases there might be some other, smaller, unit that may be appropriate.

We assume a fixed set of samples is under consideration. Assume there is some fixed threshold parameter *C*, which gives a fixed set of location calls. To fix ideas we could have a number *N* of breast cancer hybridizations and *C* could simply be a cutoff for red to green (normalized) intensity log ratio. Alternatively, we might estimate null distributions for each probe *c,* possibly with a battery of normal/normal hybridizations, and take the cutoffs as 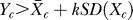 , for gain and 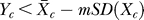 for loss, for some choices of *k* and *m*. *Y_c_* is the log ratio value for probe *c* and 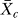 is the average value for probe *c* and *SD*(*X_c_*) is the standard deviation of probe *c* over the set of normal/normal hybridizations. We allow *k ≠ m* due to the potential lack of symmetry between gains and losses. While our implementation only contains a limited number of methods for making calls by probes, a user can apply our algorithm using any such method.

**Figure 5:**
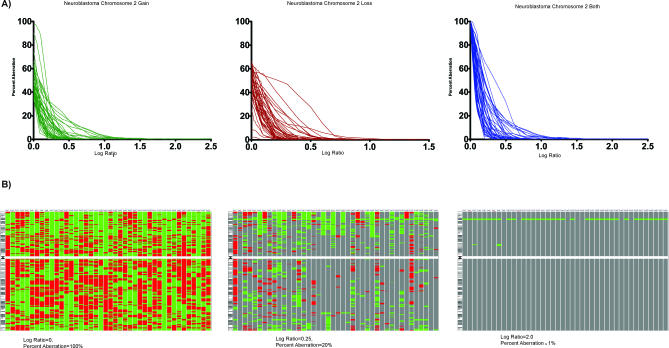
Example of the Multiple Sampling Approach on Neuroblastoma Chromosome 2 Data (A) The distribution of aberrations is plotted versus the threshold cutoff of the log ratio for each sample. The red plot represents the percent aberration of loss, green is gain, and blue is the total percent aberration at a cutoff. (B) An image of the gains and losses called at three cutoffs is shown along with the log ratio used to determine gain and loss calls and the average percent aberration at that cutoff.

A conservative value of the cutoff *C* is calculated at which there are relatively few calls being made for that value of *C,* denote this value by *C*_max_ (Figure 5). STAC is then executed on the data obtained by making calls using each of the values:

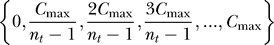



The lower the value of the threshold, the more signal and noise is involved. In our implementation, the minimum value of *n_t_* is 3, and the default value is 9. At each step we execute STAC to obtain concordance *p*-values. We subsequently perform a Bonferroni type correction, where we correct some values higher than 1/*n_t_,* and some values lower (details given in the next section). The corrected *p*-values are then reported.

**Figure 6:**
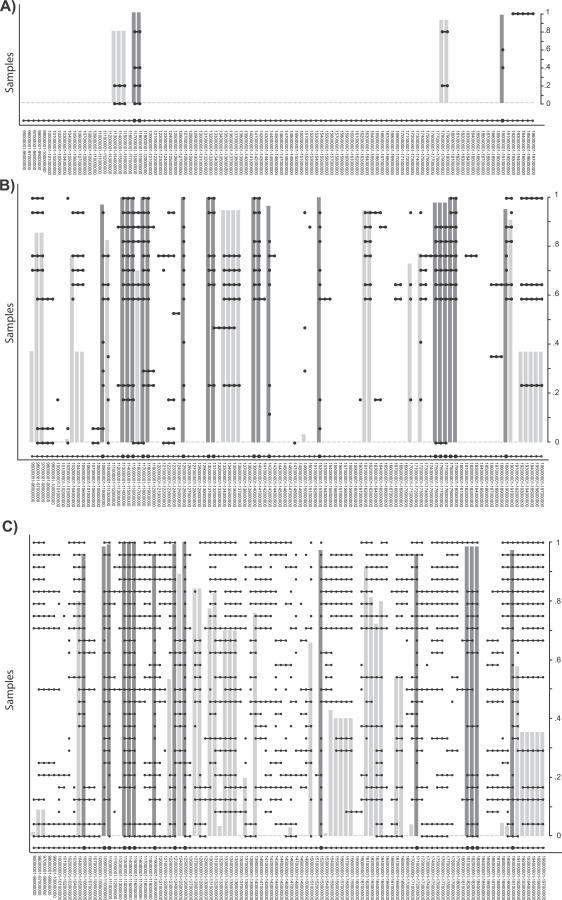
STAC Confidences for the Three Most Extreme MSA Test Values This illustrates, on real data, that one cutoff value that reveals signal in one region can obscure real signal in other regions. (A) At a high cutoff it is possible to find tight concordance across positions 117–119 Mb and 190–191 Mb. (B) A middle cutoff preserves tight concordance at 117–119 Mb but loses 190–191 Mb and picks up additional regions such as 175–180 Mb. (C) At the lowest cutoff, the aberration at positions 117–119 Mb and at 175–180 Mb are obscured by noise. However, a new region at 181–184 Mb is detected. The height of the bar corresponds to the confidence level (1 − *p*). Dark gray bars are significant with *p* < 0.05.

If every position is aberrant then no region will be significant. Therefore, at our most liberal value we are allowing excessive noise and so do not expect to detect much signal. However, if there were a strong concordance of a very weak signal we would still detect it at this level. The benefit of sampling over various values is that the tight concordance that can be found at the most liberal value may not be found at more conservative values and vice versa (Figure 6).

We explored the possibility of finding an optimal single value of *C* that maximizes the signal to noise in some overall sense, however we found that information is generally lost whenever a single value of *C* is used. This method instead provides a way of optimizing the value of C for each position of the genome independently.

### Assessment of Concordant Aberration

We describe a correction scheme that corrects the *n_t_* tests differently. This is done to balance the beneficial effect of performing tests with more cutoffs, against the detrimental effect of having to make too strong a Bonferroni correction. By prioritizing the regions we can mitigate the conservativeness of the Bonferroni correction at certain test values.

Since we are performing *n_t_* tests for each probe, we must perform a multiple testing correction. We use a modified Bonferroni correction, which requires *n_t_* to be of the form 2*^k^* + 1 for some *k*. The correction factor is based on bisecting the interval [0,*C*_max_] *k* times with varying correction factors. We then multiply the permutation *p*-values of each step by the appropriate correction factor. Specifically, we multiply those values of *C* that are introduced in the *i*th bisection by 2*^i^* ^− 1^. This gives *n* “adjusted” *p*-values *p*_1_,…, *p_n_.*. Let p* = min(*p*_1_,…, *p_n_*). If there is no aberration at the location, then the unadjusted *p*-values are uniformly distributed and

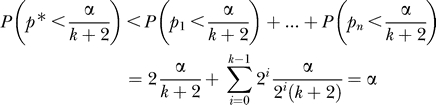



Therefore, if 
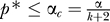
 , or 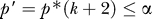 , we reject the hypothesis that the concordance at the region is due to chance with Type I error rate *α*. All MSA reported *p*-values are these corrected *p*-values, so as to facilitate comparison to a standard *α* level directly. We will refer to the multiple testing corrected *p*-value, denoted *p*′, as *p* for the remainder of the manuscript.

The varying correction factors allow us more power than a Bonferroni on our three most representative tests. This is done because we expect that any strong signal not present in any of the other values could still be significant following adjustment.

The power of this approach depends on an appropriate number of permutations being used in the analyses. If one uses only 100 permutations, then the minimum possible uncorrected *p*-value will be approximately 0.01 and if only three tests are used the minimum possible corrected *p*-value is approximately 0.03. Therefore, it is important to ensure a suitable permutation distribution based on the number of tests to be used.

### Making Single Sample Calls Using Multiple Sample Significances

The method described above reports regions and confidences measuring significant concordance. However, there is still a need to make single sample calls in order to test such questions as association between types and determination of subtypes, clustering, and other downstream analytical tests, as well as for visualization purposes. Since we are interested in conserved effects we determine the single sample calls using the information provided from multiple samples. By using the different cutoff for each region given by the cutoff that maximizes the concordance confidence, we determine the tightest multiple sample concordance for that region. These highest confidence calls are interesting because they minimize the probability of making a false single sample call while using the information from multiple samples to finely resolve single sample calls. This gives a view of the data that has all noise and nonconcordant signal removed, revealing just the concordant signal. The single sample calls work well in determining known aberrations and differences between samples, as is seen in the examples below.

### Examples

#### Formalin fixed paraffin embedded samples.

We examined a set of human breast cancers that were laser-microdissected from archived formalin fixed paraffin embedded (FFPE) tissue. These samples represent an important resource; however, they also represent a challenging aCGH application, as they tend to have significant amounts of noise. Because archived FFPE cancers and other tissues represent a vast and rich research resource, an accurate and robust analytic approach to profiling them is extremely valuable. Our goal was to use known aberrations within these samples as a benchmark for determining the ability to differentiate between sample noise and real signal.

In order to differentiate between aberrations due to the processing steps, we hybridized a set of normal–normal samples where in one channel we laser microdissected normal cells from FFPE tissue processed identically to our cancer cells. The other channel was hybridized with a universal reference from a pool of genomic DNA samples. The test samples consisted of 20 ductal carcinoma in situ (DCIS) and 23 lobular carcinoma in situ (LCIS) microdissected samples, hybridized with the same universal reference pool as the normal controls.

#### DCIS.

**Figure 7:**
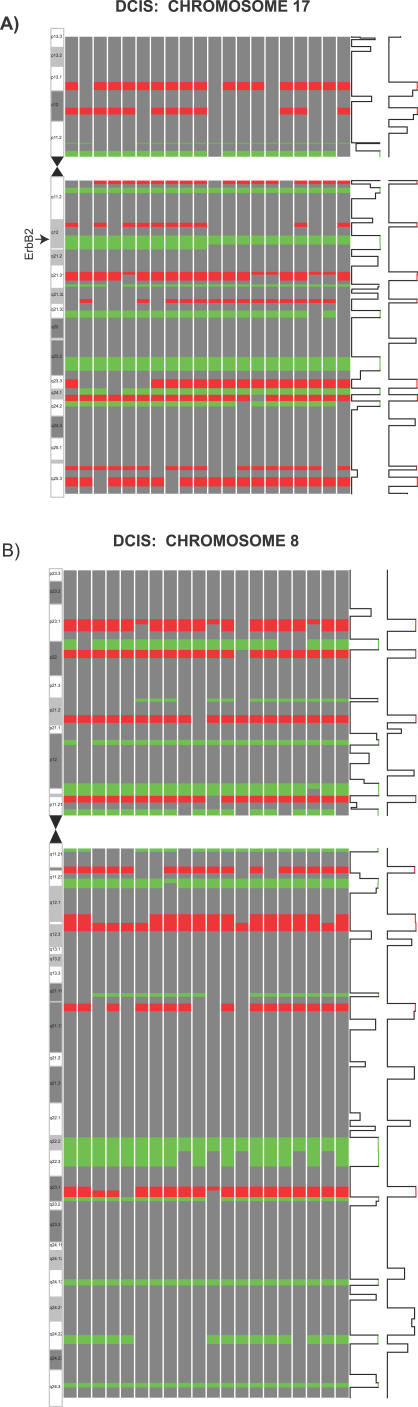
Concordant Aberrations on Chromosomes 8 and 17 in DCIS (A) Copy number change for DCIS samples. No change (grey), deletion (red), or gain (green) are indicated. Only significant aberrations are visualized. ERBB2 amplification on Chromosome 17 across 20 DCIS samples is indicated. The aberration is localized to an approximately 1-Mb region across the samples. The line graph on the right tracks the confidence at each location, where regions of significant gain are indicated in green and significant loss are indicated in red. (B) Chromosome 8 across 20 DCIS samples. A large number of losses are detected on the p arm as well as the centromeric side of the q arm, while the telomeric end of the q arm contains many gains. These general patterns are interrupted by gains on the p arm and losses on the q arm that are detected with high confidence across multiple samples.

We ran our algorithm on the 20 DCIS samples to generate confidence values for each region. This resulted in many known regions of aberration as well as other, uncharacterized regions. One known small region of amplification is on Chromosome 17q12, which involves the ERBB2 oncogene [23], aberrant expression of which is believed to occur due to genomic amplification of this region [7]. This is a small highly concordant amplification, usually limited to a region of 1–2 Mb, an area covered by only one or two probes on this array. MSA is able to identify the amplification (*p* = 0.0069) over this small region (Figure 7A). Furthermore, we were able to identify the single sample values at which this aberration occurs. We verified this amplification using immunohistochemistry staining, which confirmed the amplification in these samples.

For comparison we also utilized two single sample methods, ChARM [24] and CBS (DNAcopy) [14]. We found that on some arrays ERBB2 aberration was detected and on others it was not. However, the samples with detected 17q12 amplification did not localize the affected region to 1–2 Mb but rather indicated a much larger span that covered most of the chromosome (Figure S1). This example is indicative of the small but important regions that can be missed or mischaracterized by single sample approaches. While the amplification of this region may look like noise in one sample, when we look across multiple samples and find tight concordance we are able to identify it.

**Figure 8:**
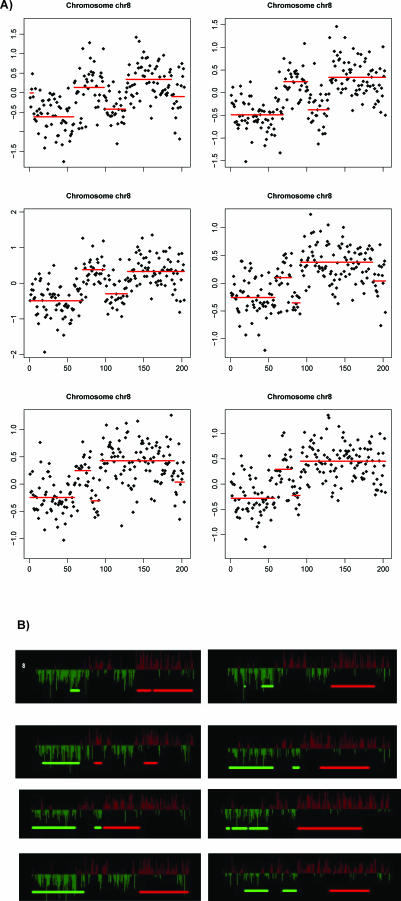
Comparison of Two Single Sample Methods on Chromosome 8 for DCIS (A) DNAcopy (CBS) indicates gross level aberration. On most samples, CBS finds a large loss on the p arm and gain on the q arm. Additionally, on some samples CBS finds a large deletion on the q arm near the centromere. The *y-*axis represents the log ratio of the sample, the *x-*axis represents the genomic position, and the red lines represent the average copy number for each segment. (B) ChARM similarly finds gross level aberration including loss of the p arm and gain of the q arm. (red, gain and green, loss). ChARM misses the amplification of MYC in many samples, and in a few samples detects the amplification as a contiguous segment covering the entire q arm. The *y-*axis represents the log_2_(T/R) ratio of the sample and the *x-*axis represents the genomic position of the probe. Red boxes signify significant gain segments, green boxes signify significant loss segments, and the height of the bars represents the log ratio.

We next compared Chromosome 8 aberrations between our method and the single sample methods mentioned above. In DCIS and invasive ductal carcinoma, Chromosome 8 has been shown to contain a large deletion on the 8p arm and many gains on the 8q arm [3,6,7,23]. MSA identifies many positions on the 8p arms that are, in fact, deleted and is able to detect differences in deletion frequencies for specific regions on the arm (Figure 7B). Rather than characterizing the entire arm as deleted, MSA identifies more precise regions of deletion that are significantly concordant across the samples. In some of these samples, the single sample methods could not pick up the deletion and in others, where a deletion was detected, it was represented as loss of most of the arm (Figure 8). We also found deletions on the q-arm, as well as localized amplifications. Finally, we were able to characterize the 2-Mb amplification corresponding to MYC amplification (*p* = 0.027) in 14/20 samples (Figure 7B).

MSA revealed a 2-Mb deletion on the 8q arm (*p* = 0.0028) as well as other smaller 1-Mb regions (*p* = 0.044–0.0028) of deletion that were previously uncharacterized. Recently, a study examining Chromosome 8 in invasive ductal carcinoma cell lines using high resolution Chromosome 8–specific tiling arrays was able to detect these same regions of deletion on the 8q arm [25]. Our analysis detected these effects even though some of them are represented by only a single array element. This demonstrates the ability of MSA to map regions at the native resolution of the array. A frequency plot of all of the DCIS significant concordant aberrations are presented in Figure S2).

#### LCIS.

We analyzed 23 cases of LCIS, another subtype of in situ mammary carcinoma, and found 733 regions of aberration. Some of these regions correspond to known aberration patterns, such as the loss of CDH1 on Chromosome 16q22.1, that are believed to be characteristic of LCIS. In fact, many pathologists use this as a discriminating marker between LCIS and DCIS [6,23,26]. In addition to localizing the deletion of CDH1 (*p*′ = 0.0028), we were able to identify many high-confidence deletions in the 16q arm (Figure S3) [27]. Chromosome 16q loss has been well-characterized within LCIS using metaphase-based CGH analysis [3,23,26]. We also identified many losses on Chromosome 16p, another characterized aberration from metaphase based CGH studies (Figure S3) [28,29]. A frequency plot of all of the LCIS significant concordant aberrations are presented in Figure S4.

#### SNP data results.

We have also tested our method on a set of publicly available T cell lineage acute lymphoblastic leukemia (T-ALL) samples [30]. This set contained 50 samples profiled on the Affymetrix 250K SNP array [30]. We performed this analysis to show that our algorithm works well on higher resolution arrays. Running in parallel with 22 nodes (one for each chromosome), the entire analysis took less than 48 h to complete. We were able to identify known, verified regions of aberration in this data as well as additional uncharacterized aberrations. A *p*-value plot is presented in Figure S6.

### Comparison to STAC Results

We applied MSA to a publicly available neuroblastoma dataset generated by Mosse et al. [31]. This data was previously analyzed using STAC based on a single processing into aberration calls [16]. We analyzed this data using nine tests each with 2,000 permutations. MSA found 747 significant regions (*p <* 0*.*05) (Table S1). In order to accurately compare the results of Diskin et al. to the MSA results, we ran STAC on the data using 2,000 permutations and applied our extension scheme and data processing steps. We selected ratio cutoffs used by Diskin et al., where a clone was called gain if the ratio exceeded 1.2 and loss if the ratio was less than 0.8. We executed STAC at this cutoff and compared the results to the MSA generated significance values. MSA was able to characterize 486 regions that STAC alone failed to detect. The single STAC run was able to detect 87 regions that MSA missed, and there were 261 regions found by both analyses (Table S2).

**Figure 9:**
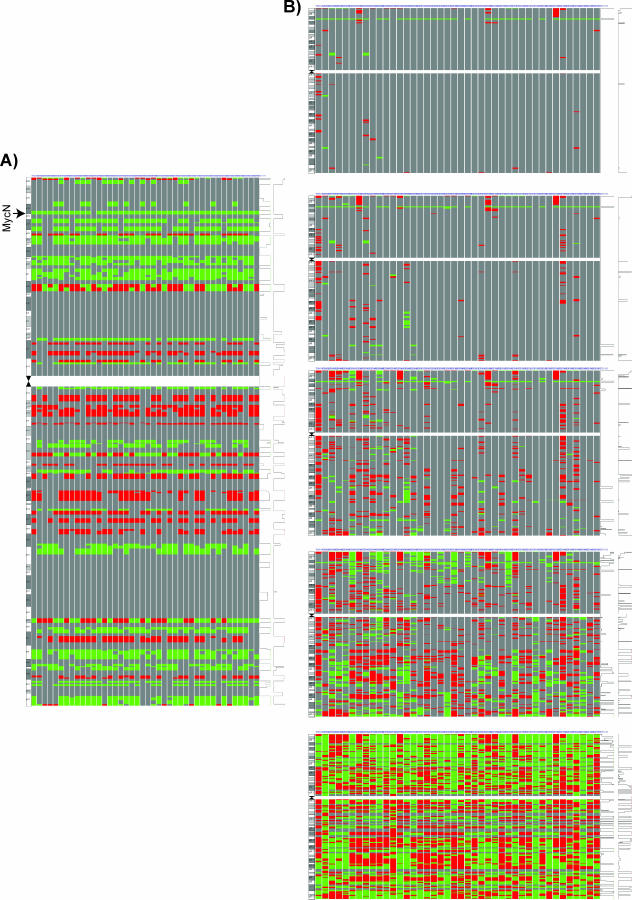
MSA Analysis of Neuroblastoma Dataset for Chromosome 2 (A) The merged view combines results from all cutoff values used. In the merged view, only the concordant aberration has been retained. All noise and nonconcordant signal has been filtered out for a clear visual representation of the concordant aberration and the contributing samples. (B) Individual views indicate results for five of the MSA test values along with their significance at each value.

Chromosome 2 represented a large number of the novel regions and we therefore decided to look at the cutoff values at which MSA determined these confidence values. The complete MSA confidence view on Chromosome 2 is plotted (Figure 9A), along with five of the MSA values (Figure 9B). There are particular values of the cutoff at which regions of tight concordance occur across the multiple neuroblastoma samples, and this concordance is no longer present at many other cutoffs. In fact, the two chromosome arms have quite different aberration patterns and limiting the analysis to one value will almost certainly lose information for one of the arms of Chromosome 2, despite their separate analysis. Therefore, by varying our cutoff and independently testing each chromosome arm (as the unit of analysis), we can detect many regions of tight concordance and high confidence. A frequency plot of all of the significant neuroblastoma aberrations are presented in Figure S5.

### Merging Single Sample Approaches into MSA

As an alternative to the strategy that makes calls at the level of the single array element, we also incorporated the CBS [14] algorithm into the MSA scheme, using CBS to determine the single sample calls and then calculating the MSA confidences for each region. A segment, based on the CBS algorithm, is a region that is significantly different from its neighboring regions [14]. Each segment has an associated segment mean (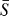 ) that represents the average value of the probes within that segment. However, segments alone are not biologically meaningful, since it is possible to have a significant segment where the segment average is less than the cutoff value for a one-copy amplification, 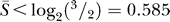 . There are many possible ways to determine aberrations from the segmentation data. One is to use threshold cutoffs, similar to those discussed earlier. For example, a segment will be called amplified if 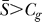 and a segment will be called lost if 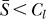 . As before, it is difficult to define a single *C_g_* and *C_l_* for all regions assayed. Furthermore, there is an additional complication in using a segmentation scheme since we must also decide on a value for the segmentation parameter *α*. If we decide on *α* = 0.01 (the default value), we will detect very few segments; however, if we increase the value of *α* we will detect more segments until, if we set *α* = 1, we will pick up almost every element as an individual segment. Therefore, we need to adjust both the value of *α* as well as the value of the threshold parameter for determining aberration.

**Figure 10:**
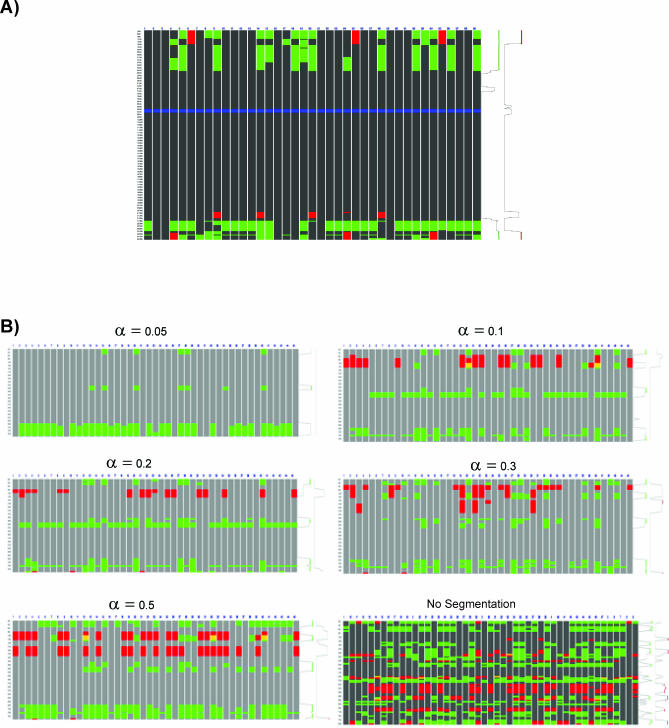
CBS Algorithm Combined with the MSA Approach The CBS algorithm was applied to segment the data and MSA was run on the resulting segments to determine conserved aberrations. Various values of the parameter for calculation of the segments were used. (A) represents the data of Mosse et al. on Chromosome 2 for the parameter value *α* = 0.05 and applying MSA. (B) represents the data of Naylor et al. on Chromosome 17 for various values of the parameter (*α*) from 0.05 to no segmentation.

We found that as we modify the threshold values for which we make calls we are able to characterize gross level aberration, but the finer-level aberrations are not detected. This loss of resolution was expected due to the loss of resolution within a single array that occurs due to segmentation. We tested this method on the data of Mosse et al. [31] and the results are shown in Figure 10A. We also varied the value of *α* to show the relative performance of our method using more liberal single sample values for the segmentation. The results are shown on the Naylor et al. [32] data using Chromosome 17 as an example (Figure 10B). We similarly applied a single sample method to the T cell leukemia 250K SNP array and then ran MSA; the results are shown in Figure S7.

MSA can be applied to segmented data to assess the significance of aberrations across multiple samples. Since most single sample methods produce continuous ratio data for segments, MSA can find meaningful aberrations that might not be found using a fixed threshold. However, performing segmentation can reduce the resolution of the aberrations and eliminate concordance across samples. While we still pick up many of the same aberrations when running MSA on segmented data, the resolution is grosser than the known aberration interval (Figure 10A). Additionally, there are aberrations that can be concordant across multiple samples but have lower amplitudes or small widths, which will prevent them from being detected by single sample methods. If these aberrations are seen across multiple samples, MSA can assign significance to those regions that might not be present post-segmentation. We find that there are many high-confidence regions that are detected by MSA in the T-ALL data that are missed when run post-segmentation.

### Simulations

#### Multiple sample copy number aberration data simulator.

We generated random datasets with known parameters to test the accuracy and specificity of the MSA algorithm. By adjusting these parameters, we tested the effects of noise and nonconcordant signal in the data on the ability of MSA to detect concordant aberrations. The model is necessarily simpler than real data; in particular, copy number and width of aberrations are fixed and the noise is constant across the entire genome. However, the model is sufficient to systematically test how the power of MSA decreases as noise and aberrant signal is increased.

We use the single sample simulator as defined, as in Olshen et al. [14] and Lai et al. [12], where each position's simulated log ratio is given by *X_i_* = μ*_i_* + ɛ for 1 ≤ *I* ≤ *G,* where ɛ ∼ *N*(0, *σ* ^2^), μ*_i_* = *cI*{*l < I < l + k*}, *G* is the genome length, *I* is an indicator random variable, *c* is the log of the copy number of the aberrations, *l* is the location of the aberration, and *k* is the width of the aberration [14]. The technical noise is controlled by the parameter *σ* ^2^.

To extend this simulator to model multiple sample concordance, we introduce additional parameters. We determine a number of samples (*n*) over which to test and then pick locations where concordant aberrations will be placed. We then specify the underlying frequency of aberration across the multiple samples for each location of concordant aberration. Finally, we specify the underlying copy number of the concordant aberrations at each location.

**Figure 11:**
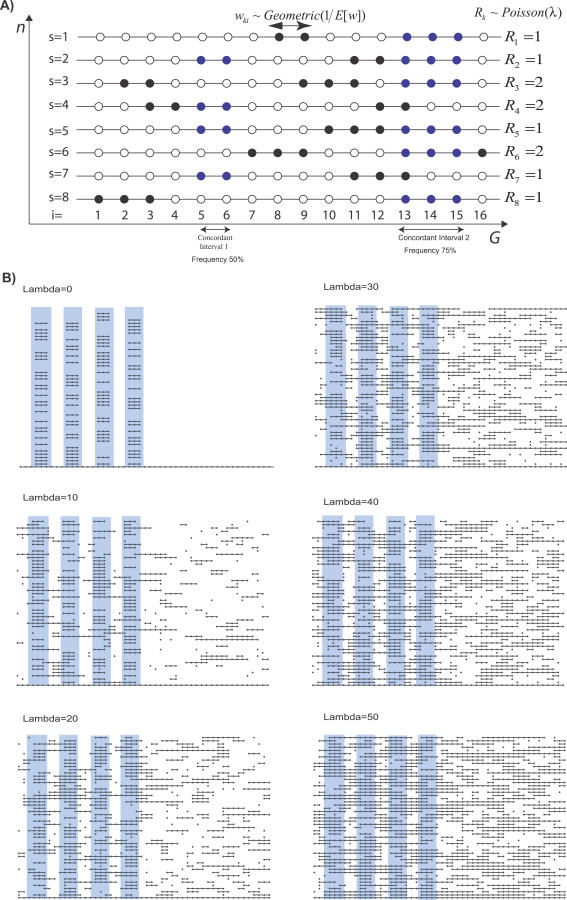
Underlying Model of the Simulated Data (A) Illustrates how concordant and nonconcordant aberrations are placed in the data. White circles represent locations containing no aberrations. Black circles represent intervals of nonconcordant aberrations. Blue circles represent intervals of concordant aberrations. In the blue and black circles the indicator random variable would have a value of 1 and the white circles would have a value of 0. The underlying frequency controls the expected number of aberrant samples containing a given concordant aberration. All circles represent random variables with the noise distribution described in the text. (B) Shows the underlying model on real data. The technical noise is not shown; only aberration intervals placed in the data are shown. The blue boxes highlight the placed concordant aberration regions. All the parameters for the different images were identical with the exception of the λ parameter, which was varied from λ = 0, …, 50.

After the underlying state is determined, we specify regions of nonconcordant aberration. We model aberrations that, while biologically real, are random from sample to sample and therefore should not contribute to multiple sample concordance. We randomly pick locations, widths, and copy number of nonconcordant intervals and they are generated using the single sample model. An illustration of the simulation method is shown in Figure 11A.

The number of nonconcordant intervals for sample *k,* denoted *R_k_,* is determined by *R_k_ ∼ Poisson*(λ). The width of the nonconcordant interval *i* in sample *k,* denoted *w_ki_,* is determined by *w_ki_ ∼ Geometric*(1/*E*[*w*]). The underlying aberrations for a model containing four concordant aberrations with varying nonconcordant aberrations are shown in Figure 11B.

#### Simulated datasets.

We generated datasets with a fixed copy number for both the concordant aberrations and the background aberrations. We note that while real data probably does not follow this assumption, for the sake of testing, this assumption causes us to be more conservative. This is because if the underlying copy number mean is higher for concordant aberrations, then we are likely to pick them up above the level of noise in our first few tests. The background noise would then be minimal and the concordance would be evident from the overlap. However, by modeling the copy number means as identical we expect to have both concordant and nonconcordant intervals in all of our tests because their underlying distributions are identical.

Datasets were generated simulating a single “chromosome arm,” because permutations and test values are calculated for each arm independently. We placed 50 markers representing nonoverlapping regions of the arm. We randomly simulated the widths of selected aberrations from one to five. The underlying frequency of concordant aberrations varied from 50% to 75% of samples. The datasets were simulated with 50 samples each. The boundaries of concordant aberrations were changed randomly by the placement of nonconcordant intervals. We also fixed the variance (*σ* ^2^) for all datasets to *σ* = 1. We estimated the true variance within multiple datasets to be approximately *σ* ≈ 0.2, which was the value used by Olshen et al. [14]. We followed the model and parameter choices of Lai et al. [12], so the results can be compared directly to their multiple comparisons.

We generated a total of 700 simulated datasets and estimated the true positive rate (TPR) and false discovery rate (FDR) overall for all the experiments. The TPR measures the number of known concordant regions that were detected as concordant aberrations by our algorithm divided by the total number of known concordant aberrations (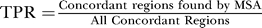 ). The FDR is the number of regions known to be nonconcordant that were called concordant by our algorithm (false positives) divided by the total number of predicted concordant aberrations by the algorithm (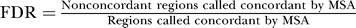 ). The signal-to-noise ratio (SNR) is defined as the mean of the segment distribution divided by the standard deviation of the segment distribution (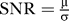 ).

#### Simulation results.

**Figure 12:**
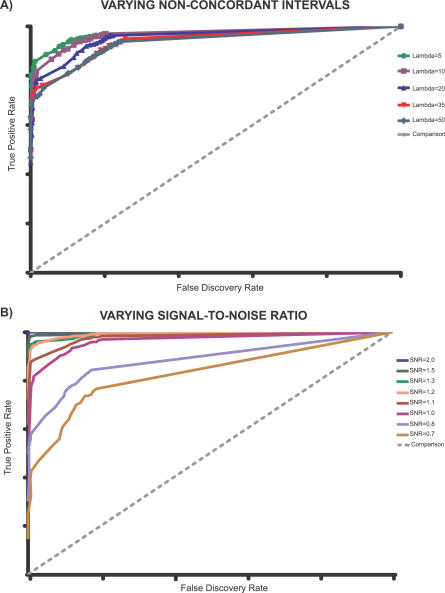
Simulated Data Accuracy Curves Receiver operating characteristic–type curves are presented here as a measure of the accuracy of the MSA algorithm. The *x*-axis represents the FDR and the *y*-axis represents the TPR for each dataset. The graph was generated by determining the TPR and FDR at selected *α* values. If *p* < *α*, then the region is called significant, and if the region is known to be aberrant it is counted toward the TPR. If it is not aberrant, it is counted as a false positive. The values of *α* from which the plot was generated are plotted and the general curve is overlaid. (A) SNR is set equal to 1 for all comparison and the amount of nonconcordant signal is varied. Lambda is the mean nonconcordant signal in each profile. As we raise the amount of nonconcordant noise, we reduce the ability to detect true signal. (B) Lambda is fixed at a value of 10 and the SNR is varied to determine the effect of changing this parameter on detection of concordant aberrations. As we decrease the SNR, it becomes harder to detect concordant aberrations. At a SNR = 2 we detect 100% true positives with almost no false positives.

We simulated data based on the model presented above. The performance of the MSA algorithm on datasets with varying SNRs is shown in Figure 12A. We found that as we increased the SNR, the sensitivity (true positive rate for a given false discovery rate) increased. Furthermore, we found that random noise does not significantly affect the results so long as the SNR is constant. As we increased the nonconcordant noise, the sensitivity decreased; the performance for varying parameters is shown in Figure 12B. However, despite increasing nonrandom noise, MSA still performs consistently well. Additionally, the FDR and FWER are controlled for all noise levels, and the specificity remains high for reasonable FDRs (<0.05) (Table 1).

**Table 1:**
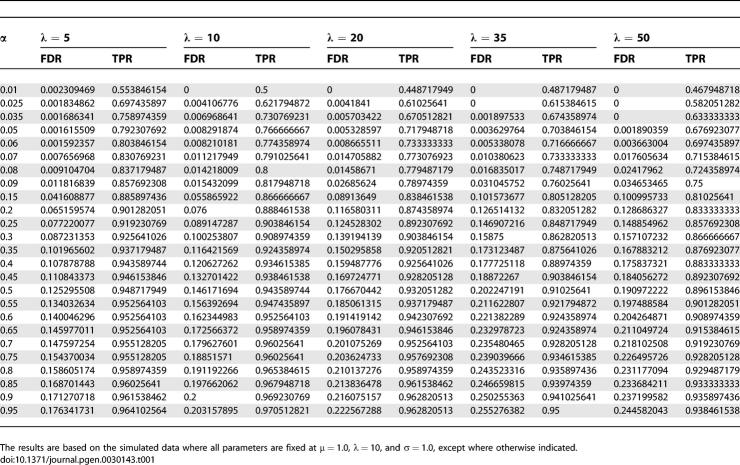
Values of the TPR and FDR for the Cutoff Values Indicated

**Table 1:**
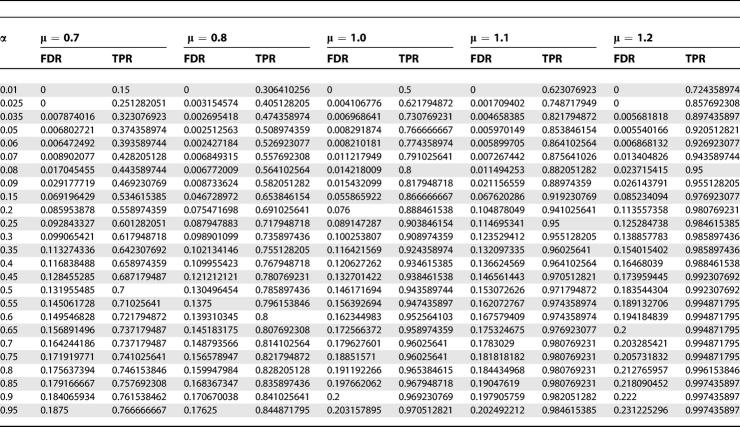
Extended.

**Table 1:**
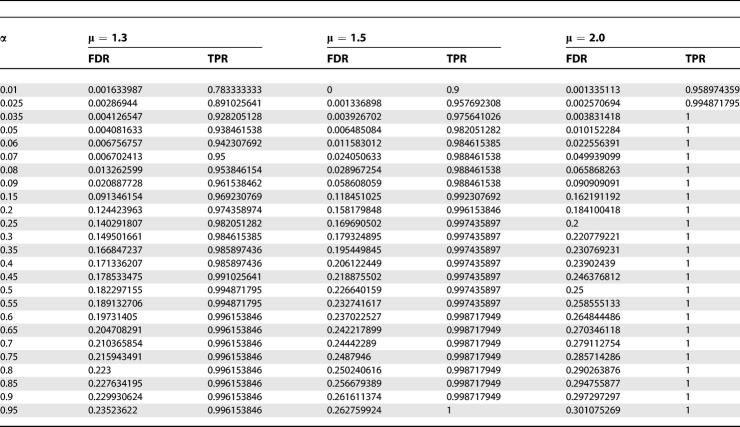
Extended.

Similarly, we varied the copy number averages and compared the affect on the specificity and sensitivity (FDR versus TPR). MSA identified 99% true positives for less than 5% FDR for an SNR of 2. MSA also identified 80% true positives for an FDR of less than 5% for an SNR of 1. These results are based on simulations where the non-random noise was varied from very low to very high (λ = 0, …, 60). If we fix a value of λ and vary the noise parameters, we see that for moderate noise parameters the accuracy and specificity of an SNR of 1 becomes better. For λ = 5 we observe 87% true positives for less than 5% FDR, and for λ = 10 we observe 81% true positives for less than 5% FDR. The specific values of the TPR and FDR are provided for each value of *α* in table 1.

#### Comparison with single sample methods.

An analysis of single sample methods was performed by Lai et al. [12], and receiver operating characteristic curves were plotted for those methods. The single sample simulator used in this manuscript follows the same model. The accuracy and specificity of MSA as compared to the single sample methods is apparent when comparing these results to Figure 2 of Lai et al. [12]. While most single sample methods performed well with high SNR, and large width of aberration, these methods all perform significantly worse for smaller SNR and widths. Our true segments were simulated with widths 1, 2, and 3 for the purposes of the characteristic curves. The ability to detect smaller regions at lower SNR becomes apparent. At SNR = 1 and width 5, the TPR of the best single sample method is approximately 40% for a false positive rate of 5%, whereas most methods fail to detect anything at all [12].

Finally, we are plotting the TPR versus the FDR rather than the false positive rate. This is because the false positive rate is not appropriate for assessing error rates in highly parallel multiple testing problems where there are relatively few false null hypotheses as compared to the total number of hypotheses. The FDR is widely accepted as the appropriate way to assess error rates in such situations. The more conservative nature of the FDR for false results in the prediction set makes us even more confident in the ability to detect smaller aberrations at lower SNR ratios than the best single sample method.

#### Observations.

We found that large amounts of simulated technical noise in the system (σ^2^) do not affect the results. Rather, the overall SNR can dramatically change the results as shown in figure 12b. Furthermore, we found that the amount of nonconcordant noise in the system can alter the detection of concordant aberrations. However, even with large percentages of the profile distorted by nonconcordant aberrations it is possible to detect most of the concordant aberrations (Figure 12A).

Additionally, we found that the width of concordant intervals did not affect the accuracy of the method. The accuracy and specificity did not change for concordant intervals of width 1, width 5, width 25, or width 100. However, the ability to pick up smaller (width) aberrations at lower underlying frequencies decreased as we increased the nonconcordant noise in the system. Additionally, we found that the underlying frequency of aberration can change the specificity dramatically and increase the effects of the other variables. With high SNR we can detect aberrations with lower underlying frequency. As we decrease the SNR, a higher underlying frequency is needed to detect concordant aberrations. Similarly, as the nonconcordant intervals increase, the harder it becomes to detect smaller frequency aberrations.

Additionally, the number of samples included in the simulation model can affect whether we detect regions as aberrant. As we increase the number of samples, it becomes easier to detect concordant aberrations. The larger the number of samples the more likely we are to detect lower underlying frequency aberrations within a dataset. However, very low frequencies become harder to detect regardless of the number of samples. As we decrease the nonconcordant noise, we can begin to detect smaller frequencies with greater accuracy.

The length of the simulated genome can affect the results detected. If we increase the size of the genome and keep the number of aberrations and background the same, we find that specificity and accuracy increase. This result is not surprising, as there are more possible arrangements of the null data and the likelihood of overlap in the null model is smaller. This increase in specificity and sensitivity seems to be the same change that is observed with the increase in the number of aberrations per model.

The width of aberrant intervals does not affect the performance of the algorithm. The only effect seems to come from the percentage of the genome that is aberrant, similar to the effect of increasing the genome size. If we fix all other parameters, as we increase the width of intervals the sensitivity decreases in a similar fashion. However, if we have a larger genome size and wider aberrations, we see no decrease in sensitivity.

In real data with high background aberration it is possible to pick up low frequency events (<5%). However, in our simulation model we found that the underlying frequency of aberration can affect the results that are detected. For very low frequency of aberration (<5%) it is hard to detect concordant aberrations in our simulation model. If we increase the SNR, there is a minimal increase in the specificity and sensitivity. Similarly, if we decrease the nonconcordant intervals or increase the number of samples, there is a minimal increase. However, if we model the concordant noise and nonconcordant noise with different underlying means, we can begin to detect lower frequencies with greater sensitivity and specificity. In reality, the means of concordant and nonconcordant intervals are probably different, although we do not have a biological understanding of this difference to accurately model it. We note that this is a potential explanation for our ability to detect low frequency aberrations in real data.

## Discussion

We demonstrate a powerful multiple sample approach for the analysis of array-based comparative genomic hybridization data and illustrate the effectiveness of this method in detecting known small regions of aberration at the native resolution of the arrays, with high statistical confidence. Aside from the detection of known regions of aberration, we have also identified many uncharacterized aberrations. The power in the method relies on the use of liberal single sample methods together with a permutation-based statistical test for analysis of concordant genomic regions.

In theory, even if there is an “optimal” single sample cutoff value for making aberration calls, there may still be conserved regions of aberration that are not detected. Even though at lower levels there may be more noise in the data, we are not more likely to pick up false signals because STAC accounts for the higher rate of random aberration. While MSA approaches the problem of determining interesting regions across multiple samples rather than within a sample, we can use the results of MSA analysis to determine the singe sample values for each experiment. This also acts as a valuable visual aid.

### Biological Motivation

The method presented in this manuscript assesses the significance of these aberrations as characteristics of a defined class of samples. This is done by looking at each location of the genome and determining the probability of the concordance occurring across the samples, as compared to the background rate of aberration. This reveals regions that are conserved due to a nonrandom pressure as compared to the background rate of genomic aberration. Therefore, if a genomic aberration does not contribute to the overall fitness of the cancer, it is unlikely to be conserved across samples at a rate greater than the random rate of aberrations in the samples. In this way, the method attempts to model a known biological phenomenon in a robust statistical manner.

### Interpreting the Results

MSA provides adjusted *p*-values for significance of aberration. The null hypothesis we are testing is the absence of concordant genomic aberration at position X. Therefore a significant result indicates that there is evidence for concordant aberrations at position X. This is not to say that there are no aberrations at nonsignificant locations, but rather that there is no significant concordant aberration. This is in contrast to segmentation, or single sample methods, described earlier. In reality, an aberration may be quite large, while the concordant part of the aberration is small. Therefore, one must not consider an MSA region of gain or loss indicated in a sample as representing the total length of the aberration in the sample. Our method aims to identify only the conserved segment of this aberration.

MSA can detect conserved heterogeneity within a subgroup as small as two samples. This can be seen in some of the results provided in this manuscript. However, our method does not always detect such subtle effects; the exact results depend on the rate of aberration in the genome. If there is little noise, then two samples can contribute to a significant result; however, if there is a lot of noise the same result may be indistinguishable from random concordant noise and missed.

Finally, when running MSA on a chromosome arm, it may fail to identify very large aberrations such as whole chromosome gains and losses. This is because MSA looks for significant localized concordance. The less local, the more samples might be needed to see the effect, while whole arm gains and losses will not be seen in any case, when running a single arm analysis. If one is interested in gross effects such as whole arm gains and losses, MSA can be run at the whole genome level.

### Generalizations

The method is presented as a two-channel array application with examples specifically from two-channel data. However, the method generalizes to one-channel datasets. The only difference is the methods used to determine single sample values and relative copy number aberrations without a reference ratio. In the two-channel case there is a clear gain versus loss distinction (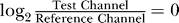 ); however, in the case of one-channel data this is not the case. There are multiple ways of avoiding this issue. If one has paired normal samples, we can form log ratios based on the test hybridization and the paired normal hybridization such that the log ratio is defined as 
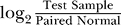
 . Alternatively, if one does not have paired samples, then a standard denominator based on a pool of normal hybridizations can be used to form log ratios.

Furthermore, we are working on extending our algorithm to detect regions of concordant loss of heterozygosity in SNP microarray data. We believe this will be a simple extension to our current approach with some modifications necessary for calculating the probabilities of loss of heterozygosity at a given position on the genome.

We have illustrated the use of MSA as a method for determining regions of conserved aberration in cancer genomes. However, this method can be used for other questions, including determining concordant copy number variations in the genome. So long as the question of interest is a multiple sample question, such as, what are the regions that contain more copy number polymorphisms than would be expected by chance? We believe the method generalizes to many areas where the underlying null model accurately tests the question of interest.

### Conclusions

We have shown the effectiveness of the MSA methodology on several datasets, each of which helps demonstrate different strengths of the method. First, we have demonstrated the ability to identify meaningful biological information that most current methods either miss entirely or mischaracterize. Second, we have demonstrated the ability of our method to distinguish between signal and noise within an extremely noisy, but important, sample resource (FFPE tissue). Third, we have demonstrated the increased power of our method over the use of a single cutoff value. Finally, we have demonstrated the ability to detect regions of aberration at high resolution.

The promise of aCGH is the ability to detect copy number aberrations with accuracy and high resolution. MSA allows for the detection of significant regions of aberration in a statistically significant manner at high resolution. MSA allows for the determination of conserved aberrations across a class of samples, which is important to accurately profile cancer and other diseases. Finally, MSA results can be useful for classifying samples, testing association between regions and tumor types, and testing for various class prediction variables.

## Materials and Methods

### Array and data extraction.

We created our 6,912-probe microarray using a human BAC clone set spaced at 1-Mb intervals throughout the genome [33]. We hybridized our samples to the array, where the reference channel consisted of a pool of degenerate oligonucleotide–primed PCR amplification products from a commercial DNA source. Aliquots of labeled target and reference DNA were cohybridized to each BAC microarray with 100 μg of human Cot-1 DNA (Invitrogen, http://www.invitrogen.com) to block repetitive sequences. The arrays, in Corning Hybridization chambers (http://www.corning.com), were incubated at 37 °C for 72 h, then washed, dried, and scanned with the GenePix Microarrray Scanner (Axon Instruments, http://www.moleculardevices.com). Data was extracted using the GenePix Pro Software package.

### Sample isolation.

A skilled histotechnologist cut and mounted single 10-μm thick paraffin sections of each target tissue onto PET-membrane slides used with the SL μCUT System (Molecular Machines & Industries, http://www.molecular-machines.com). Using conventional methods, we deparaffinized, rehydrated and stained the sections with hematoxylin. Within 1–2 h, we placed each section into a microdissection unit that sandwiches the tissue section between a clean glass slide and the membrane and microdissected it with the SL μCUT System. This precision microdissection gave us near-pure populations of LCIS and DCIS cells for whole genome scanning.

### Data normalization.

We observed an intensity dependent bias and performed print tip–specific loess normalization within each array [22]. The normal samples were similarly normalized. We did not perform scale normalization, as the distributions between the samples were comparable to each other. We used the standard deviation scheme for making gain/loss calls as described earlier.

### Normal–normal distribution.

We generated a distribution based on 23 normal mammary samples. We identified normal mammary tissue that has been previously formalin fixed and paraffin embedded and subsequently laser capture microdissected normal cells. DNA was subsequently extracted, labeled with Cy3, and hybridized to our array. Our Cy5 channel contained identical pooled genomic DNA as our sample hybridizations to allow for direct comparison.

### Published neuroblastoma data.

Previously published neuroblastoma data [31] was used to test our method. The raw data was downloaded from http://acgh.afcri.upenn.edu/nbacgh. Regions were extended and genome spacing was standardized prior to analysis using MSA. STAC analysis was conducted using the threshold parameters provided by Mosse et al. [31], Gain 1.2 and Loss 0.8.

### Genomic mapping.

For comparative purposes, all genome coordinates are based on Build 34 (Hg16 July 2003 Freeze) of the human genome.

### Published T cell leukemia SNP data.

Previously published T cell leukemia SNP data [30] was used to test our method. The raw data (CEL files) were downloaded from http://www.stjuderesearch.org/data/ALL-SNP1/ and are accessible from the Gene Expression Omnibus (GEO). The data was preprocessed and normalized using the GenePattern (http://www.broad.mit.edu/cancer/software/genepattern/) [34] modules SNPFileCreator and CopyNumberDivideByNormals [35]. The output file was fed directly into the MSA software package.

### MSA algorithm and simulation model implementations.

The MSA algorithm and the Simulation model are implemented as stand-alone java applications and are available along with documentation and technical specifications at http://www.cbil.upenn.edu/MSA. STAC v1.2 is incorporated into the MSA algorithm and is also available as a stand-alone java GUI application at http://www.cbil.upenn.edu/STAC.

## Supporting Information

Figure S1 DNAcopy (CBS) Results for Six DCIS Samples on Chromosome 17 CBS characterizes the Erbb2 amplification at a gross level over half the chromosome length. This amplification can be more finely localized to a 1–2-Mb region by MSA (see Figure 3).
(749 KB PDF)

Figure S2 Frequency Plot of Significant Aberrations in DCIS Samples For all regions of significance (*p* < 0.05) a frequency of aberration was calculated and is plotted alongside the chromosome ideogram. Green represents regions of significant gain and red represents significant loss. The width of the bar represents the length of the aberration and the height represents the frequency of aberration. The tick marks represent 25%, 50%, 75%, and 100% frequency, respectively.
(2.2 MB PDF)

Figure S3 MSA Analysis of Chromosome 16 for LCIS The ability to map aberrations at high resolution allows detection of boundaries at the resolution of the array for known but broad copy number differences on Chr16 previously identified by metaphase CGH including high resolution detection of CDH1.
(909 KB PDF)

Figure S4 Frequency Plot of Significant Aberrations in LCIS Samples For all regions of significance (*p* < 0.05), a frequency of aberration was calculated and is plotted alongside the chromosome ideogram. Green represents regions of significant gain and red represents significant loss. The width of the bar represents the length of the aberration and the height represents the frequency of aberration. The tick marks represent 25%, 50%, 75%, and 100% frequency, respectively.
(2.5 MB PDF)

Figure S5 Frequency Plot of Significant Aberrations in Neuroblastoma Samples For all regions of significance (*p* < 0.05), a frequency of aberration was calculated and are plotted alongside the chromosome ideogram. Green represents regions of significant gain and red represents significant loss. The width of the bar represents the length of the aberration and the height represents the frequency of aberration. The tick marks represent 25%, 50%, 75%, and 100% frequency, respectively.
(2.3 MB PDF)

Figure S6 Results of the MSA Algorithm Run on T-ALL SNP Data [30] A *p*-value plot of significant regions identified by MSA in the T-ALL SNP data. The dotted line on the right represent the significance level (*p* < 0.05) for gain, the left represents the significance level for loss. The results are plotted at a 100-kb resolution.
(5.1 MB PDF)

Figure S7 Results of Single Sample Method Followed by MSA Analysis Applied to T-ALL Data Profiled on a 250K SNP Array A *p*-value plot of significant regions identified by MSA in the segmented T-ALL SNP data. The dotted line on the right represent the significance level (*p* < 0.05) for gain, the left represents the significance level for loss. The results are plotted at 100kb resolution.
(4.7 MB PDF)

Table S1 MSA Significant Regions Detected in Neuroblastoma Dataset Published by Mosse et al. [31] Regions listed are all regions that were detected by MSA with corrected *p*-value < 0.05. Genomic coordinates are based on the build 16 of the human genome (hg16) based on the same data previously reported by Mosse et al. and Diskin et al. All genomic regions containing coverage, with the exception of clones on Chromosome Y, were included in the analysis.
(79 KB XLS)

Table S2 MSA Significant Regions Not Present in a STAC Analysis of the Neuroblastoma Data MSA p-values are compared to STAC p-values at these positions. MSA was able to characterize 486 regions not detected by STAC alone.
(63 KB XLS)

### Accession Numbers

The normalized and raw data (GPR files) generated in this study are accessible from the National Center for Biotechnology Information (NCBI) Gene Expression Omnibus (GEO, http://www.ncbi.nlm.nih.gov/geo/), through accession number GSE8601. The previously published T Cell Leukemia raw data (CEL files) is accessible at GEO through accession number GSE5511.

## Abbreviations

aCGH: array based CGH
CBS: circular binary segmentation
CGH: comparative genomic hybridization
DCIS: ductal carcinoma in situ
FDR: false discovery rate
FFPE: formalin fixed paraffin embedded
FWER: family-wise error rate
LCIS: lobular carcinoma in situ
LCM: laser capture microdissection
MSA: multiple sample analysis
SNR: signal-to-noise ratio
STAC: significance testing for aberrant copy number
T-ALL: T cell lineage acute lymphoblastic leukemia
TPR: true positive rate
