# Supplementary material for: Assessing the Significance of Conserved Genomic Aberrations Using High Resolution Genomic Microarrays
Source: PLoS Genet. 2007 Aug 24;3(8):e143. doi: 10.1371/journal.pgen.0030143 (PMC1950957; doi:10.1371/journal.pgen.0030143)
Supplement: Supplementary file 1 [file pgen.0030143.sg001.pdf]

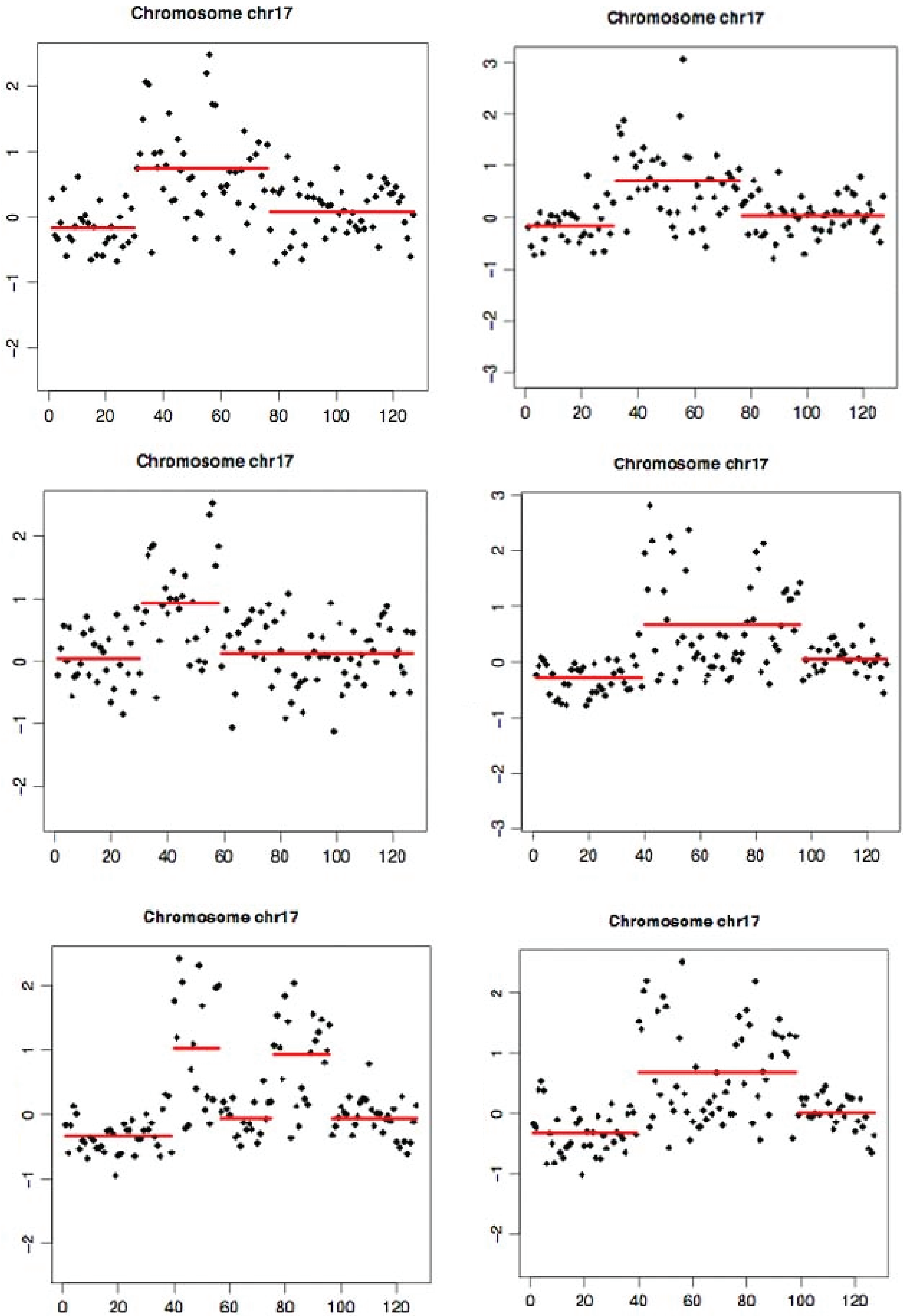
Chromosome chr17
Chromosome chr17
Chromosome chr17
Chromosome chr17
Chromosome chr17
Chromosome chr17
-2 -1 0 1 2
-3 -2 -1 0 1 2 3
0 20 40 60 80 100 120
