# Supplementary material for: Assessing the Significance of Conserved Genomic Aberrations Using High Resolution Genomic Microarrays
Source: PLoS Genet. 2007 Aug 24;3(8):e143. doi: 10.1371/journal.pgen.0030143 (PMC1950957; doi:10.1371/journal.pgen.0030143)

LCIS: Chromosome 16

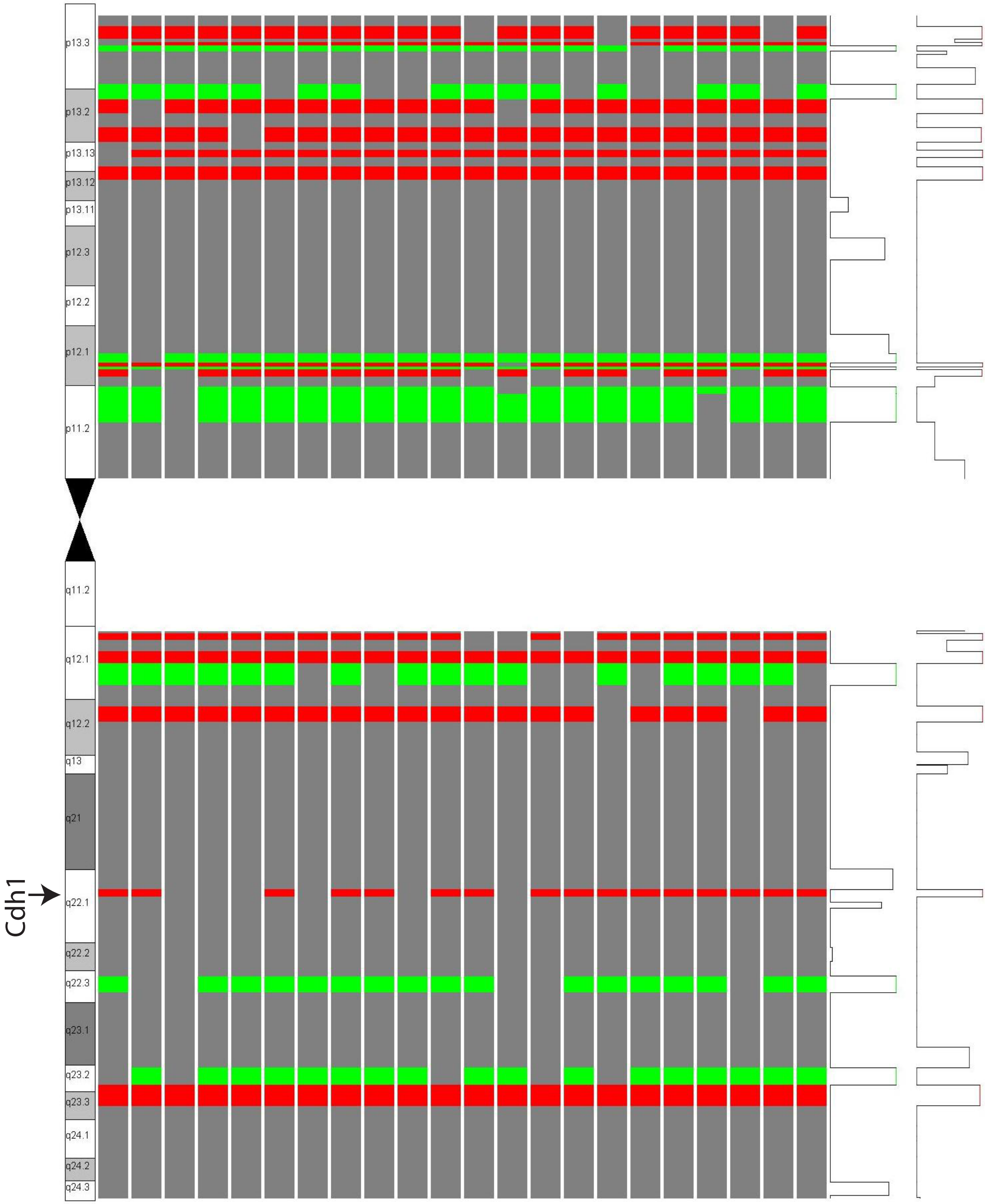

Supplement: Supplementary file 3 [file pgen.0030143.sg003.pdf]
