# Supplementary material for: Assessing the Significance of Conserved Genomic Aberrations Using High Resolution Genomic Microarrays
Source: PLoS Genet. 2007 Aug 24;3(8):e143. doi: 10.1371/journal.pgen.0030143 (PMC1950957; doi:10.1371/journal.pgen.0030143)
Supplement: Supplementary file 4 [file pgen.0030143.sg004.pdf]

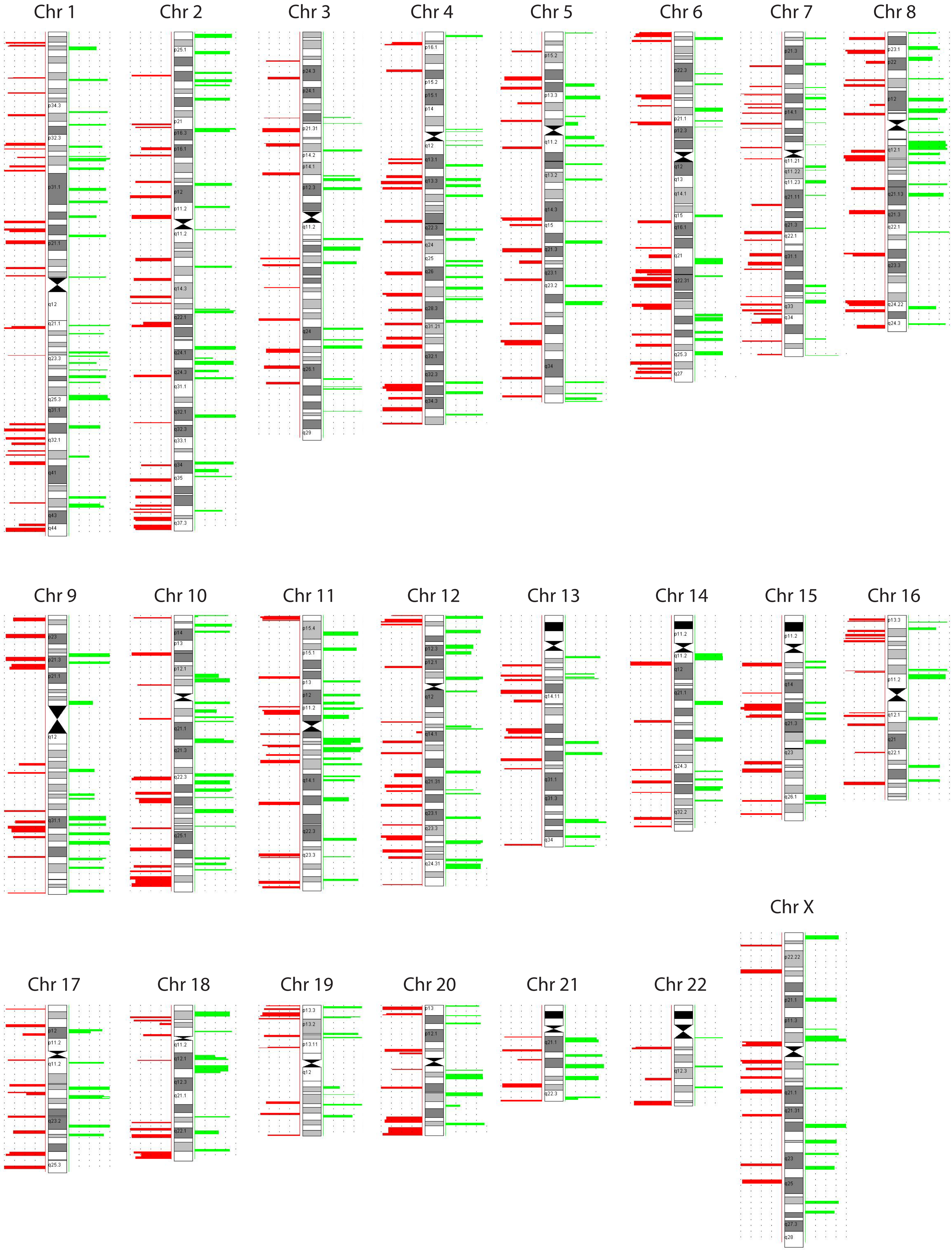
Chr 1
Chr 2
Chr 3
Chr 4
Chr 5
Chr 6
Chr 7
Chr 8
Chr 9
Chr 10
Chr 11
Chr 12
Chr 13
Chr 14
Chr 15
Chr 16
Chr 17
Chr 18
Chr 19
Chr 20
Chr 21
Chr 22
Chr X
