# Supplementary material for: Assessing the Significance of Conserved Genomic Aberrations Using High Resolution Genomic Microarrays
Source: PLoS Genet. 2007 Aug 24;3(8):e143. doi: 10.1371/journal.pgen.0030143 (PMC1950957; doi:10.1371/journal.pgen.0030143)
Supplement: Supplementary file 7 [file pgen.0030143.sg007.pdf]

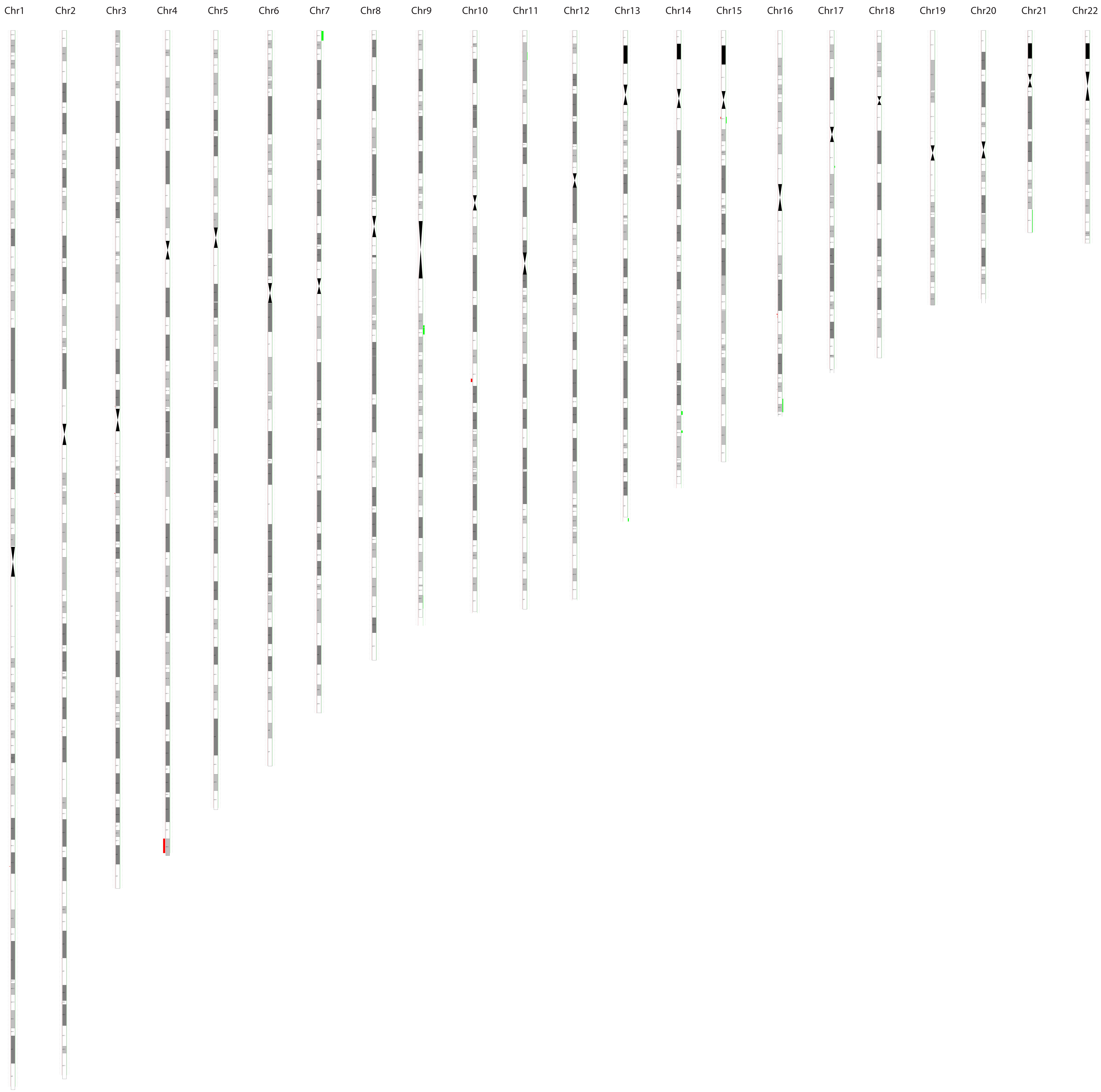
Chr1
Chr2
Chr3
Chr4
Chr5
Chr6
Chr7
Chr8
Chr9
Chr10
Chr11
Chr12
Chr13
Chr14
Chr15
Chr16
Chr17
Chr18
Chr19
Chr20
Chr21
Chr22
